# AFM-Nano Manipulation of Plasmonic Molecules Used as “Nano-Lens” to Enhance Raman of Individual Nano-Objects

**DOI:** 10.3390/ma12091372

**Published:** 2019-04-27

**Authors:** Angélina D’Orlando, Maxime Bayle, Guy Louarn, Bernard Humbert

**Affiliations:** 1 INRA BIA UR 1268, 44300 Nantes, France; angelina.dorlando@inra.fr; 2 IMN J. Rouxel, UMR 6502 CNRS-Univ Nantes, BP 32229 44322 Nantes, France; maxime.bayle@cnrs-imn.fr (M.B.); guy.louarn@cnrs-imn.fr (G.L.)

**Keywords:** enhanced Raman spectroscopy, plasmonic nanoparticles, AFM-nanomanipulations, optical near-field, plasmonic molecules

## Abstract

This paper explores the enhancement of Raman signals using individual nano-plasmonic structures and demonstrates the possibility to obtain controlled gold plasmonic nanostructures by atomic force microscopy (AFM) manipulation under a confocal Raman device. By manipulating the gold nanoparticles (Nps) while monitoring them using a confocal microscope, it is possible to generate individual nano- structures, plasmonic molecules not accessible currently by lithography at these nanometer scales. This flexible approach allows us to tune plasmonic resonance of the nanostructures, to generate localized hot spots and to circumvent the effects of strong electric near field gradients intrinsic to Tip Enhanced Raman Spectroscopy (TERS) or Surface Enhanced Raman Spectroscopy (SERS) experiments. The inter Np distances and symmetry of the plasmonic molecules in interaction with other individual nano-objects control the resonance conditions of the assemblies and the enhancement of their Raman responses. This paper shows also how some plasmonic structures generate localized nanometric areas with high electric field magnitude without strong gradient. These last plasmonic molecules may be used as "nano-lenses" tunable in wavelength and able to enhance Raman signals of neighbored nano-object. The positioning of one individual probed nano-object in the spatial area defined by the nano-lens becomes then very non-restrictive, contrary to TERS experiments where the spacing distance between tip and sample is crucial. The experimental flexibility obtained in these approaches is illustrated here by the enhanced Raman scatterings of carbon nanotube.

## 1. Introduction

The strong demand to miniaturize to improve the spatial resolution and to decrease the detection limits in optical spectroscopic devices has initiated a significant amount of research in the field of the plasmonics [1,2,3,4,5]. The recent propositions in the literature are based on the nano-structuration of nanoparticles (Nps) of metal (Reference [5] and references therein). Indeed, electrons on a metal surface, excited by electromagnetic fields (for instance by focusing light beams on Nps), are able to enhance the electric field locally up to several orders of magnitude. The spatial area where the field is “concentrated” is as small as just a few nanometer squares which provides the opportunity to design the electric field of light with a spatial resolution of 2–3 orders of magnitude below the diffraction limit. This effect has been at the origin of the Surface Enhancement Raman Spectroscopy SERS spectroscopy and of Tip Enhanced Raman spectroscopy (TERS) that presents an exponential decrease. The enhancement was predicted to be increased on to originate in the near-field generated in inter-particle gaps, for instance within the colloidal aggregates [1,2,3,4,5]. However, because the experiments involved wide distributions of aggregates of different sizes and shapes or of many different nano-structured surfaces, the correlations between their near-field properties and SERS spectra were highly challenging. Then many experiments have tried to rationalize these nano-structuration by lithography, colloidal depositions, self-similar assembling [5,6], often in a “top-down” way. In the field of molecular fluorescence detections, the combination of the process assembly simplicity with the fluorescence enhancement made the self-assembled colloidal nanoparticle gap antennas optimal to extend a wide variety of single-molecule applications toward the micromolar concentrations [7]. This self-assembly may be helped by a pre-step of lithography [8]. Thus, for instance, spatially programmable Au nanorod dimers have been obtained with enhanced capacities for fluorescence applications, highlighting the opportunities for precise tunability of the plasmonic modes in larger assemblies. F. Koendernicks recently published a review on the different possibilities of this kind of approach to obtain single photon nano-antennas on large spatial areas [9]. These kinds of approaches have also been used to build substrates for surface enhanced Raman spectroscopy uses [10,11]. For instance, large-area multifunctional wedge and pyramid arrays directly onto planar substrates via template stripping [10] allowing in the same time to magnetically trap the species and to characterize them by SERS in the optical near-field. By lithography, substrates constituted of gold nano-stripes have been proposed and deeply studied in SERS applications [11]. The SERS efficiency of probe molecules was investigated on non-annealed and annealed lithographic gold stripes. The SERS intensity is not linked to the far-field response of the non-annealed stripes. This mismatch between the far-field and near-field response comes from a significant contribution of the surface roughness of the stripes. The annealing of lithographed substrates by decreasing roughness resulted in a strong weakening of the Raman signals. Their results further suggested that the SERS effect was more pronounced in the red part of the visible range, far from the plasmon resonance of the structures and was essentially generated by the sub-20nm structures coming from the roughness than more by the organized lithographed over 20 nm. Therefore, nano-shapes, sub-20 nm, for SERS applications remains an important key to investigate. However, another bottom-up approach was proposed, in particular by Chuntonovs’ team [12,13]. The idea was to describe small aggregates of some metallic Nps as plasmonic molecules [5,12,13]. Indeed, well-defined assemblies of Nps that sustain surface-plasmon resonances have been labelled “plasmonic molecules” [12,13,14], where the individual “atoms” of these plasmonic molecules are then the individual metallic Nps, called “plasmonic atoms”. In this paper, the individual gold Nps will correspond to “plasmonic atoms”. In the dipole approximation, the “plasmonic atoms” may be described as individual Nps dipoles and the inter-strength depends then on the single-particle polarizability, which scales as the cube of the diameter of the individual Nps. Moreover, the energy of the interaction of the two dipoles induced on the surfaces of the individual Nps upon excitation scales as the inverse of the cube of the gap-distance between the particle centers. Nordlander and co-workers developed a plasmon hybridization description [12,13,14,15,16] which predicts the modes of interacting Nps (plasmonic atoms) and calculates their corresponding energies, i.e., the resonance spectra.

In this application field of individual plasmonic molecules, we have developed since 2013 [17] an experimental approach where molecules are built atom per atom with the help of an Atomic Force Microscope (AFM) coupled directly with an optical spectrometer in order to monitor the optical changes. Tong et al. initiated this approaching 2008 to understand the enhancement of Raman signals of nanotubes of carbon (Single wall nanotubes of carbon SWCNT) by approaching an individual Au Np to one isolated target SWCNT via AFM [18]. The advantage of this method is to use the same Au Nps in all the experiments, thus all the physical changes are caused by changes of the shape of plasmonic molecule or to the distances between plasmonic atoms but never due to intrinsic Np changes (the individual diameters, individual shapes, surface chemistry are fixed). Thus the discussion of the experimental results are facilitated. The remaining experimental problem in this approach is to have the perfect control of distances between the plasmonic molecule and the interacting nano-objects. Indeed, due to the strong decrease of the optical near fields (as previously evoked in inverse of the cube of the gap distances between Nps) the signals strongly depend on the different relative spacings. Consequently, if the goal is to use one plasmonic molecule as a nano-sensor, its response varies with only spatial parameters. This paper proposes a shaping of plasmonic molecule to solve this problem and to avoid the strong gradient effect of the near field around metallic Nps. This original approach will be applied to follow the Raman spectra of individual nanotubes of carbon. This kind of object is now a “quasi” academic object widely described and used in the same kind of experimental conditions [19,20]. For instance, Reichs’ team realized the coupling of carbon nanotubes as a one-dimensional model system to lithographed gold nanodimers acting as near-field cavities. Their plasmonic cavities enhanced the Raman signal of a small nanotube bundle by three order of magnitude. Our paper will show that this enhancement may also be reached by nano-manipulating one single particle from the assembly of Au Nps constituting the cavity.

## 2. Materials and Methods

### 2.1. Materials

**Figure 1:**
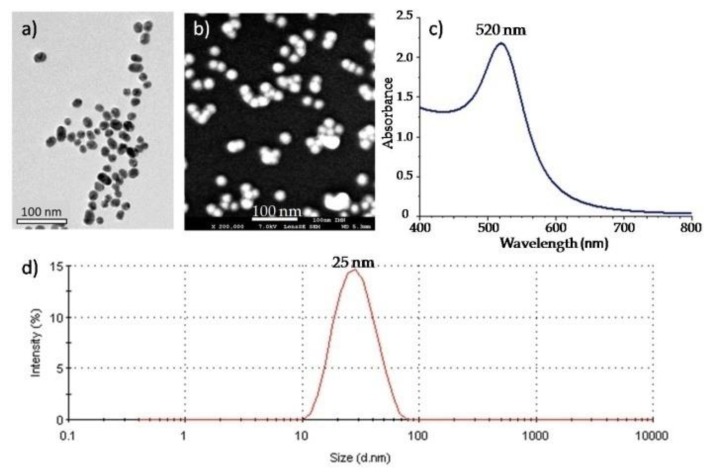
Example of characterizations carried out on Au Np colloidal suspensions synthesized during this work: (**a**) TEM images (scale bar of 100 nm) of one deposited drop on carbon sheet, (**b**) scan electronic image of deposited drops from a spray on SEM substrates (**c**) UV-vis spectrum recorded for this colloidal solution and (**d**) DLS characterization.

We synthesized AuNPs with very precise protocols ensuring that the only parameters to be modified were the reactant molar ratio (citrate to gold ratio) and the sequence of reagent addition [21]. We followed the ‘Turkevich’ protocol introduced in 1951 [22] rationalized recently by Li Shi et al. [21]. Our target for this work has been to obtain a diameter around 20 nm for the manipulations. The gold (III) mother solution was diluted with water to obtain 50 mL of "yellow orange" solutions at [AuIII] = 0.25 mM in 100 mL double-necked round flasks. The flasks were immersed in an oil bath to maintain the temperature around 95 °C. Then 2.5 mL of a citrate solution (molar ratio fixed 2) preheated during 10 min at about the reaction temperature were all added at once. After 15 to 20 minutes of reaction, the solutions were then cooled slowly to the room temperature. These colloidal suspensions were characterized by dynamic light scattering (DLS), ultraviolet visible (UV-vis) absorption and electronic microscopy. Respectively, DLS experiments were carried out using on a NanoZS apparatus (Malvern Panalytical, Malvern, UK) operating at k = 632.8 nm; UV–vis spectroscopy was performed on Cary 5000 Scan UV-Visible Spectrometer (Agilent, Santa Clara, CA, USA) and the primary size distributions and the shape of particles were determined by transmission electron microscopy (Hitachi H-9000 NAR, Tokyo, Japan, operating at 300 kV with a Scherzer resolution of 0.18 nm). Our dispersion were then characterized by a single modal distribution around 22 nm ± 5 nm. Figure 1 displays an example of a series of characterizations carried out on one of our synthesizes.

Purified arc-discharge (>90%) SWCNTs retreated through three temperature cycles under Argon atmosphere, allowing removal of residual amorphous carbon and repair of some defects, were used in our experiments. Ultra-sonic baths were used to increase the probabilities to obtain individual SWCNTs, before being drop casted on a clean mica substrate. No surfactants were used. To avoid the spread of water on the substrate, which might be an inconvenience during nano-manipulation, we had to control the relative humidity (RH), keeping it around 35% RH and at 22 °C. Finally, the Raman spectra of SWCNT sample, were recorded and compared to the Resonant Raman fingerprints of the free standing well index-identified SWCNTs.

### 2.2. Experimental Methods 

Intermittent contact mode imaging and nanotubes manipulations are carried out with a NanoWizard® AFM (JPK Instruments, Berlin, Germany). For these experiments, a standard rectangular cantilever (PPP-NCL-W probes, Nanosensors, Neuchâtel, Switzerland is used with a free resonance frequency of 250 kHz and a typical spring constant of about 40 N/m. 

Raman spectra were recorded with a triple subtractive monochromator T64000 Horiba-JobinYvon (Kyoto, Japan) spectrometer equipped with an Olympus confocal microscope with a motorized 80 nm step XY stage. The spectrometer was also equipped with a notch filter to eliminate the Rayleigh scattering with a 100 cm^−1^ cut-off. The detector was a CCD cooled by liquid nitrogen. The samples were excited with either an argon laser line at 514.53 nm or with a 561 nm solid laser or yet with a Helium-Neon laser at 633 nm. The details of the experimental setup are given elsewhere [23]. The laser beam with a controlled power was focused on samples with a spot size diameter of about 0.8 µm. The Raman backscattering was collected through the objective of the microscope (×100, numerical aperture of 0.95 or ×20 numerical aperture of 0.35) and dispersed by a 1800 grooves·mm^−1^ gratings to obtain 2.7 cm^−1^ spectral resolutions for the 514.53 nm excitation beam and around 2.5 cm^−1^ with 561 nm excitation. The wavenumber in vacuum accuracy was better than 0.8 cm^−1^. The polarization response of the optical device was checked by measuring the depolarization ratios for the perfectly known bands of reference liquid pure products. For instance, the experimental depolarization ratio for the 459 cm^−1^ symmetric component of the CCl_4_ spectrum is 0.02 to 0.005 for the different wavenumber positions of the centered CCD camera or 0.03 to 0.01 for the C–H stretching mode of CH_2_Cl_2_. During all experiments, the signal of a single crystal of silicon has been systematically checked. The results displayed here were obtained in such a way that the setup worked in the confocal microscope mode that was aligned with the automated XYZ table. To improve spatial and spectral resolutions, appropriate gratings according to the excitation wavelength and confocal mode were used. The focused power of the laser beam was checked for each wavelength and for each sample to avoid any transformation or heating of the samples. Here, only the spectra obtained at the limit of detection (defined here as the ratio signal on noise equal or superior to 3) will be displayed in order to discuss and compare the order of magnitude of enhancement of our results. The enhancement effects in near fields were too high to use the same focused laser power to estimate these comparisons; the usual power used to record conventional Raman spectra applied for all nanostructures damaged indeed either SWCNT or plasmonic molecules. Typically, if a power at about 1 mW was used for conventional Raman, only few micro-watt powers had to be used in the resonant nanostructures.

### 2.3. Numerical Modeling

For each experimentally studied structure, 3D finite element (FE) calculations were performed using COMSOL software (Multiphysics version 4.5), with Radio Frequency (RF) and Wave Optics modules adapted to the electromagnetic waves affecting objects at the nanoscale. Our calculations were beforehand confirmed by comparing numerical results with analytical results obtained in the Mie’s theory field. Dimensions of each modeled Au Np were based on the corresponding AFM topography images. The environment surrounding nanoparticles has been assimilated to the air (with an optical index of 1.00015).

The absorption and scattering cross sections are defined by the rate of electromagnetic energy W that is absorbed or scattered across the surface of an imaginary sphere (centered on the particle) divided by the incident irradiance P_inc_ (W/m^2^):

(1) $$\sigma_{\mathrm{abs}}=\frac{W_{\mathrm{abs}}}{P_{\mathrm{inc}}}{\;\mathrm{and}\;\sigma }_{\mathrm{diff}}=\frac{W_{\mathrm{diff}}}{P_{\mathrm{inc}}}$$

With $W_{abs}$ total absorbed power rate (W), obtained by the integration of the energy loss $Q_{\mathrm{loss}}$ (W/m^3^) in the particle volume $V_{p}$:

(2) $$W_{\mathrm{abs}}=\underset{V_{p}}{∭}Q_{\mathrm{loss}}\;\mathrm{dV}$$

and ${\;W}_{\mathrm{diff}}$ the total scattered power rate [W], obtained by the integration of the Poynting vector of the scattered field $P_{\mathrm{dif}}$ (W/m^2^) on the surface of a virtual sphere around the particle, between air domain and a Perfectly Matched Layer (PML):

(3) $$W_{\mathrm{diff}}=\underset{S}{∯}P_{\mathrm{dif}}.\;n\;\mathrm{dS}$$

where n is the unit normal vector to the virtual sphere. The $Q_{loss}$ parameter is calculated by COMSOL.

Finally, the far-field variable, calculated on the internal boundary of the PML, is defined by:

(4) $$E_{far}=\;\underset{r\to \;\infty }{\lim}\left(r\;E_{diff}\right)$$

The far-field variable $E_{far}$ represents the scattering amplitude rather than the physical electric field, and is measured in units [*E*_far_] = (m·V/m) = (V) [24].

Having only one medium and relatively simple structures, the direct resolution generates the total field, whose incident field can be subtracted when processing the results. In almost all of the calculations in this work, the amplitude of the incident field is taken at 1 V/m. The dielectric properties of gold are derived from those proposed by Etchegoin, Le Ru and Meyer [25].

### 2.4. Combining Raman and AFM-Manipulation

**Figure 2:**
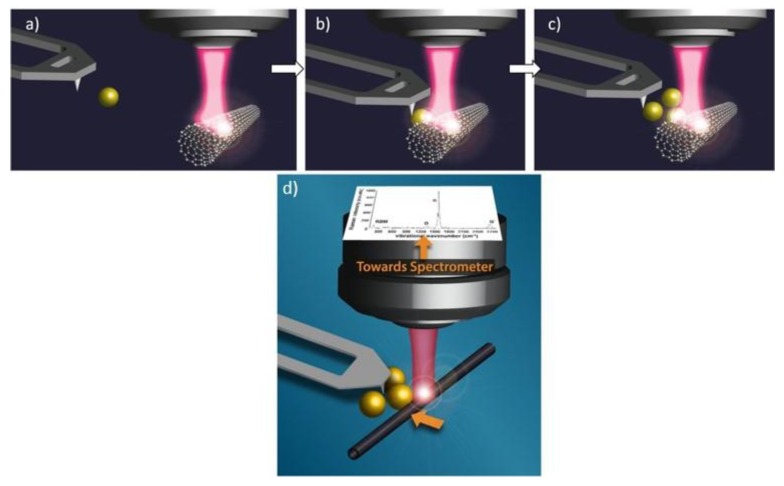
Scheme of our experiments of Raman-AFM Manipulation combining. (**a**) Manipulation of the first individual Au Np, coming from the reservoir; (**b**) Approach of the SWCNT by the Au Np pushed by the AFM tip; (**c**) Designing of the nanostructure (plasmonic molecule) with three plasmonic atoms and (**d**) Monitoring by Raman spectroscopy at several wavelengths of excitation.

The objects studied in this work were obtained by deposing a 20 µL drop of CNTs suspension followed by the drop of a second 20 µL volume near to the first area of deposition. Thus small "reservoirs" of Au Nps were constituted not far from the area where some CNTs have been deposited. In a first step AFM images were collected to find isolated individual CNTs or small bundles of CNTs. In a second step, some nano-manipulations have been undertaken on the CNTs themselves, for example to separate two individual CNT objects. The last steps consisted in Au Nps nanomanipulations: from the Au Np reservoirs, one by one Au Np was driven in the near field of the chosen CNT in order to design the shapes or the relative spacing inter the plasmonic molecule in interaction with the selected CNT (Figure 2).

**Figure 3:**
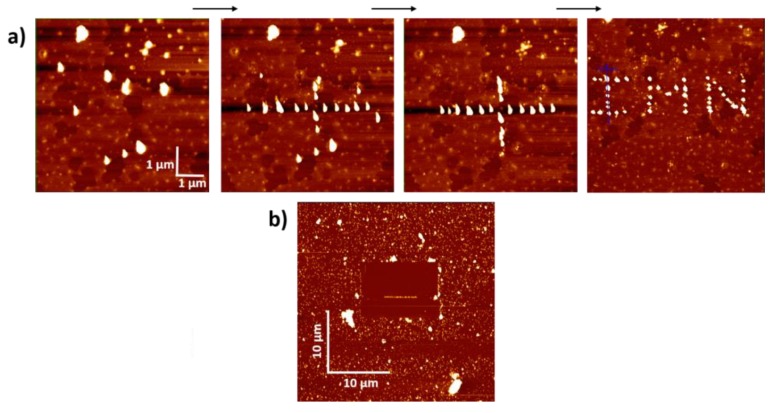
Examples of gold structures created by AFM manipulation; (**a**) different steps leading to a cross, then to the IMN logo; (**b**) long gold NP line of about 6 μm, the surrounding area being cleaned by the AFM-Tip in contact mode. (bar scales at top are 1 µm and down 10 µm).

Figure 3 displays examples of the kind of structures that we built in less than one hour for each one of them, on areas of around 5 × 5 µm^2^, i.e., larger than the focused laser beam used in optical measurement. Thus for this paper, via the AFM manipulation of gold nanoparticles, the plasmonic nanostructures of interest were constituted such as the visible cross or line in Figure 3, with always the same Au Np individuals. We were thus able to adapt the plasmon modes according to our wishes via the hybridization of the plasmonic modes, as mentioned in the previous part, avoiding the much used lithography process which is less precise at the nanometer scale. Numerous two-dimensional geometries are thus conceivable.

Before Au Np nano-manipulations, the Raman signals at different excitation wavelengths were recorded to characterize the optical response of the selected CNT. If the wavelength corresponded to a possible electronic transition of this CNT, a spectrum was measurable (Raman Resonance), but when the wavelength did not match with a characteristic transition of the CNT individual (the general case) no spectrum was detectable. After Au Np AFM manipulations but before the optical studies of the nano-structurations, the investigated area was cleaned of other gold particles and of any dust particle surrounding the tubes by the AFM-tip scanning in contact mode. Thus the cleaned area was consistent with the spatial area probed by optical microscopy (Figure 3b). Since the same AFM-tip was used to manipulate and to image at each step the area of interest, the tip was progressively damaged resulting in sometimes AFM image artifacts. For example, Figure 3 presents the deformation of the spherical shapes of Au Nps in prismatic shapes after 500 nano-manipulations for writing “IMN”. Then in this case the spatial resolution has been degraded from better than 1 nm to higher than 3 nm.

## 3. Results

**Figure 4:**
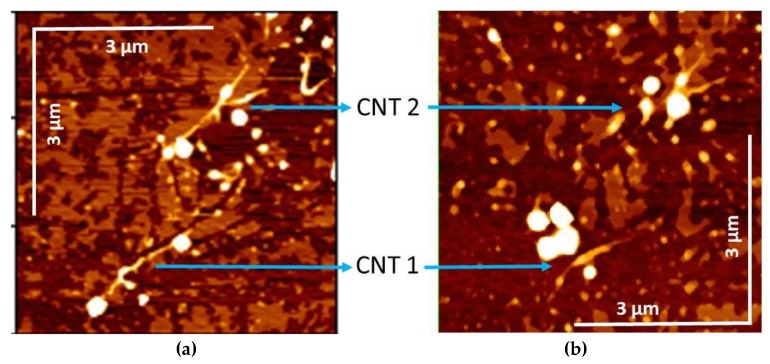
(**a**) Example of an AFM image of two isolated CNTs (or small bundles of SWCNTs) in presence of dispersed Au Nps in the surrounding. This area was after cleaned of dust and gold particles surrounding the SWCNTs to obtain the configuration displayed at right. (**b**) The same SWCNTs embellished with some Au Nps plasmonic molecules.

**Figure 5:**
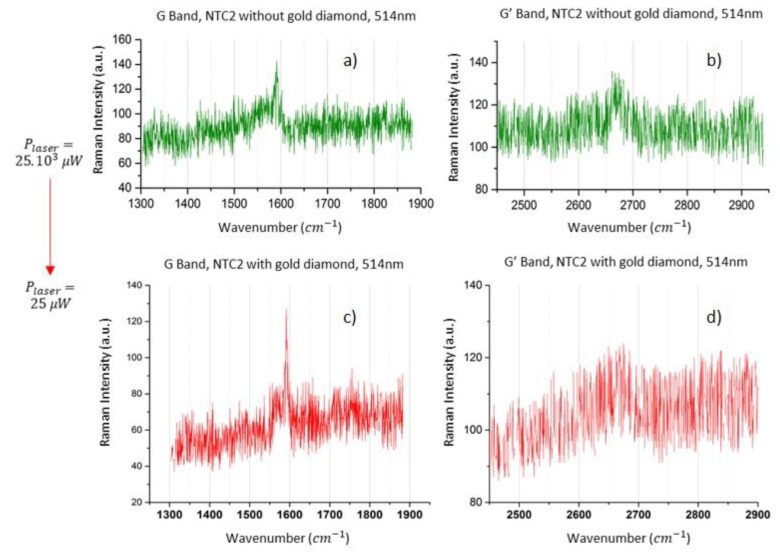
Spectra obtained (6 accumulations of 10 s) at the limit of detections, for the CNT 2 tube of the Figure 3; (**a**,**b**) G and G’ bands of CNT 2 without gold excited at 514.53 nm, for a significant focused power of 25 mW, on around 1 µm^2^; (**c**,**d**) the Raman signals at the detection threshold of CNT 2 dressed with two gold particles on each side, recorded with an incident laser power of 25 µW, i.e., 1000 times lower than in the previous conditions.

**Figure 6:**
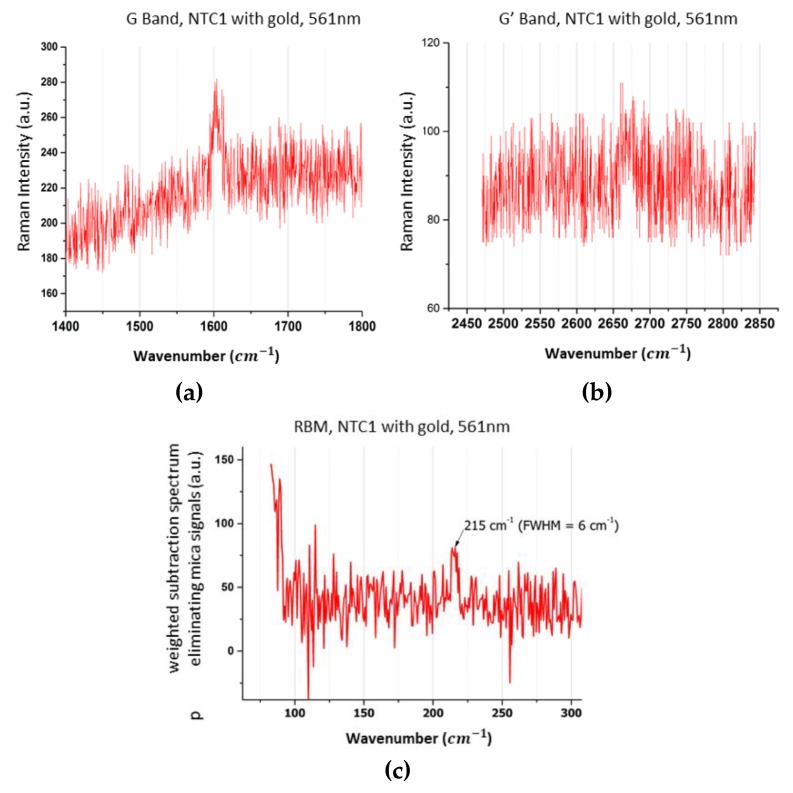
G (**a**) and G’ (**b**) bands of the CNT 1, excited at 561 nm for a low power of 3.3 μW; (**c**) RBM of the CNT 1 excited at the same power and wavelength, obtained after removing the mica substrate bands.

First, we have localized by AFM some isolated CNT on a Mica substrate (Figure 3), called CNT 1 and CNT 2. According to the AFM measurements, these CNTs whose heights are less than 3 nm are almost individual CNTs (at most, small sets of two to four individual CNTs). Then gold structures such as the diamond in Figure 4 have been created around these CNTs. Raman spectroscopy analysis have then been done before and after this gold manipulation step with the same wavelengths. Raman spectroscopy of the CNT 2 and CNT 1 are presented respectively in Figure 5 and Figure 6.

### 3.1. Raman Spectroscopy Results for CNT 2

Isolated and without gold, this set (CNT 2) does not resound at 633 nm and very weakly at 561 nm. With an excitation at 514.53 nm, a Raman signal is only detected, with a “high” incident power, from 25 to 50 mW: the bands G (~1591 cm^−1^) and G’ (~2670 cm^−1^) (green curves on the top graphs of Figure 5) were displayed (with six accumulations of 10 s). Now, in the presence of two gold particles (on each side of the CNT 2—Figure 4), the signals for G band at 1591 cm^−1^, G’ band at 2670 cm^−1^ are detected with a power of only 25 µW, 1000 times lower than the previous one. The G band seems to be slightly more amplified and especially thinner than the G’ band (verified for higher incident powers at 50 µW). The measurement of the mid-height band indicates a width of about 6 to 7 cm^−1^. This last result, combined with the fact the resonant Raman spectra were obtained only for one wavelength, confirmed the hypothesis of probing one individual tube that could be stated on the basis of the AFM height measurements.

### 3.2. Raman Spectroscopy Results for CNT 1

Red and yellow (561 nm) excitations of isolated and gold-free CNT 1 does not generate any Raman scattering. Only an excitation at 594 nm with 5 mW (six accumulations of 5 s) focused around the CNT 1, gives a Raman spectrum. By contrast, when CNT 1 is embellished with a diamond-shaped gold plasmonic molecule and a single nano-particle on its other side (Figure 3b), a Raman fingerprint spectrum appeared at 561nm and under a remarkable weak focused power of 3.3 μW (Figure 6) (6 accumulations of 10 s). In this case, all the usual modes are observed: the radial breathing mode, RBM, at 215 cm^−1^, the fine G band at 1600 cm^−1^ with a full width at half maximum (FWHM) of only 6 cm^−1^, and the G’ band at 2668 cm^−1^. Even if the RBM intensity is weak here, almost hidden in noise and in many bands of mica in this spectral region, the corresponding spectrum of Figure 6a, where the RBM appeared, was obtained by weighted subtraction of the substrate signals. Thus, due to the presence of the plasmonic molecule, the enhanced Raman spectrum excited at this wavelength can become possible with an enhancement factor higher than 10^3^. This estimation based only on the ratio of detection thresholds could have appeared unsatisfactory and we have tried to compare Raman intensities collected with the same focused powers recorded in conventional configuration and with plasmonic structures. In these cases, the power (some mW) used to excite the conventional Raman spectrum, damaged the plasmonic molecules, either by moving Au Nps or by changing relative spacing or yet by trapping the Au Nps on the CNTs. Therefore in this work, we will use only the thresholds to give estimations of the near field effects obtained with the plasmonic molecules. We are developing studies on these different phenomena involving the interactions CNT-Au Nps.

## 4. Discussion

The enhancement factor of the Raman signals excited at 514.53 nm, of CNT 2 by the Au Np dimer (Figure 5) from either side of CNT 2 was estimated between 500 and 1000 from the Raman experiments. This estimation is based on the value of the ratio of the detection thresholds, collected on the same area probed on the same optical objective, with the same spectroscopic configuration. This enhancement is then produced only by the presence of the plasmonic structure. Some experiments with an excitation wavelength outside of the resonances (of CNTs and AuNps) at 785 nm have been tried to estimate the backscattering effect of the AuNp structures presence on our Raman signals. We had not been able to record any signals without damaging our structures (the Finite Element Method (FEM) simulations gave only a backscattering power outside of the resonance multiplied by around 2). Thus, we assumed that the essential effect in these experiments comes from resonance plasmonic effects. In the CNT 2 case, the hybridization of plasmonic levels of the two nanoparticles is obtained with a gap spacing of around 10 nm, between the two particles of 22 nm, without chemical contact between CNT and Au Nps. In this configuration, the numerical Comsol FEM modeling gives an enhancement of the near electric field of about 4, at 525 nm. If we assumed that the SERS signal is as the power 4 of the electric field amplitude, then the SERS will be multiplied by 256. If we compare the dimer to the monomer, the maximum of the plasmonic resonance is slightly shifted from only 5 nm and broadened by a factor 2 as described in the literature on dimer plasmonic molecules [5]. This result is comparable in particular with those obtained by Zhu et al in 2014 [26] that measured SERS enhancement factor for gap distances of 10 nm between two 90nm disks between 2 and 3 orders of magnitude. Then in the case dimer-CNT 2 assembly, the Raman measurement may be explained only by the enhancement of local electric field created by the dimer, without other important effect involving shift of resonance or coupling between structures or yet electronic transfer. However, this enhancement is strongly dependent on the gap spacing and on the position of the Raman scatterer, because the gradient of the electric field magnitude in the gap is strong. Therefore, we had proposed other plasmonic molecules to diminish this gradient effect and tested them with CNT 1.

The CNT 1 (Figure 6) trapped in the gap between the diamond shape molecule and an isolated plasmonic atom gives us other pieces of information than the CNT 2. The enhanced Raman spectrum of CNT 1 allowed us to identify its chirality nature in coherence with the no direct observed resonance effect at 561 nm of the CNT 1 without Au Nps. Indeed, few CNTs on Kataura diagrams [27,28,29] correspond to the RBM wavenumber observed for the CNT 1. The “closest CNT” on the Kataura plot of our observations would be the metallic (8,8) SWCNT characterized by a RBM wavenumber at 216 cm^−1^. It is characterized by an electronic transition of around 18,300 cm^−1^ (i.e., 545 nm), not too far of 561 nm excitation. The second kind of possible SWCNT would be the metallic (12,3) tube. This tube is characterized by its electronic transitions at around 16,500 cm^−1^ (i.e., 605 nm) and 19,900 cm^−1^ (502 nm) and whose RBM wavenumber is expected at 217 cm^−1^. These second possibility of SWCNTs is then not characterized by an electronic transition excitable at 561 nm.

The second experimental fact concerning CNT 1, is that the G band intensity, usually not very relatively strong for metallic CNTs by comparison with RBM signal, is more enhanced, with the diamond shaped plasmonic molecule than the RBM and G’ signals. The usual Raman profile is then deformed by the plasmonic resonant molecules. 

**Figure 7:**
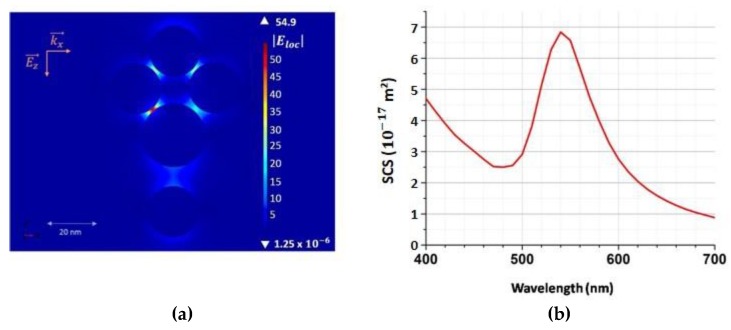
(**a**) Near-field mapping around the gold structure. (**b**) The plasmonic structure Scattering Cross Section (SCS).

The enhancement observed around 560 nm is clearly explained by the plasmonic molecule electronic structure. As displayed in the Figure 7, the computed scattering cross section of the plasmonic molecule studied here, shows a resonance at 555 nm with the incident field parallel to the gap direction. The local electric field in the gap at the resonance wavelength is multiplied by 10, i.e., the Raman scattering could be expected to be multiply by 10^4^.

Now to understand the change of profile of the Raman spectrum, the coupling between the CNT and this resonant plasmonic molecule must be investigated. Indeed, the CNT Raman resonances (see for example Reference [30]) may be obtained either at the wavelength corresponding at the electronic transition (Raman resonance at the excitation) or at the inelastic scattered wavelength (Raman resonance at the scattering). Raman excitation profiles display these two resonances [30] versus laser wavelength, resonances with the incident or with scattered photons.

**Figure 8:**
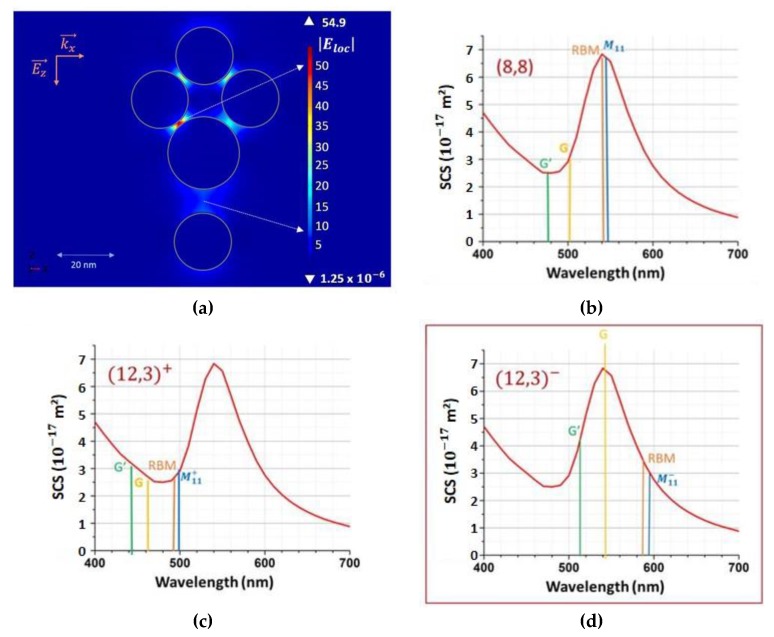
FEM computations coupled with metallic CNT behaviors. (**a**) the FEM simulations of the electric field show (indicated with the white arrows) two strong enhanced field area. The CNT 1 is in the region where the field is enhanced by 10. The different possibilities to generate Raman resonance effects (at the excitation, transition M 11 in blue or at the scattered resonances for the three principal Raman CNT signals). (**b**) If CNT 1 was a (8,8) SWCNT the resonance at 561 nm would enhance the excitation Raman process, in this case, all the spectrum would be enhanced. (**c**) For the first electronic transition of a (12,3) SWCNT, neither the transition at the excitation and no inelastic scatterings could be enhanced by the resonance of the plasmonic molecule. (**d**) For the second transition of a (12,3) SWCNT, the Raman resonance at the excitation is weakly enhanced, while the Raman resonance at the scattering would favor the G-signal. This latter case allows interpreting the signals observed in Figure 5.

Taking in account both these resonance effects of CNTs gives the principle scheme shown in Figure 8 applied to our case. In this scheme, the Raman spectra may be excited at (i) the wavelength corresponding to the energy allowing the electronic transition (blue lines in Figure 8) in order to induce the enhancement of the whole inelastic spectrum without strong change in the relative profile or (ii) also to a wavelength that could favor, not the excitation, but the inelastic scattering processes (orange and green lines in Figure 8). The first (i) condition could be favored in the (8,8) CNT and then the Raman signal would be dominated by the RBM signal with a weaker intensity for G, as usual for metallic CNT. However, that does not correspond to our observation. Now let us consider the second (ii) condition for (12,3) chirality. For example, to favor the G band, the wavenumber of the incident laser beam must correspond to the electronic transition wavenumber plus the vibration wavenumber characteristic of the G mode (here around 1600 cm^−1^). Then if CNT 1 is assigned to a SWCNT (12,3), this last inelastic amplified process will be obtained at its maximum for 16,500 cm^−1^ (electronic transition) + 1600 cm^−1^ (G mode), i.e., 18,100 cm^−1^ (corresponding to 552 nm). In our experimental case, we have obtained this kind of enhancement with an incident wavelength at 561nm. If we add that the Raman fingerprint is deformed with a G band more enhanced, we assign the observed enhancement to a Raman resonance effect obtained with inelastic scattered photons induced by the plasmonic enhancement at this resonant wavelength due to the peculiar shape of the diamond plasmonic molecules. If the experiment was conducted to look into the resonance at the inelastic scattered G’, then the plasmonic molecule would have to be deformed to shift its plasmonic resonance to 510 nm. The experimental Raman observation of Figure 5 is then only interpreted by the enhancement effect produced by the diamond plasmonic molecule coupled with the single plasmonic atom dressing the (12,3) CNT, where the signal G is favored by a resonance at the inelastic scattering energy.

**Figure 9:**
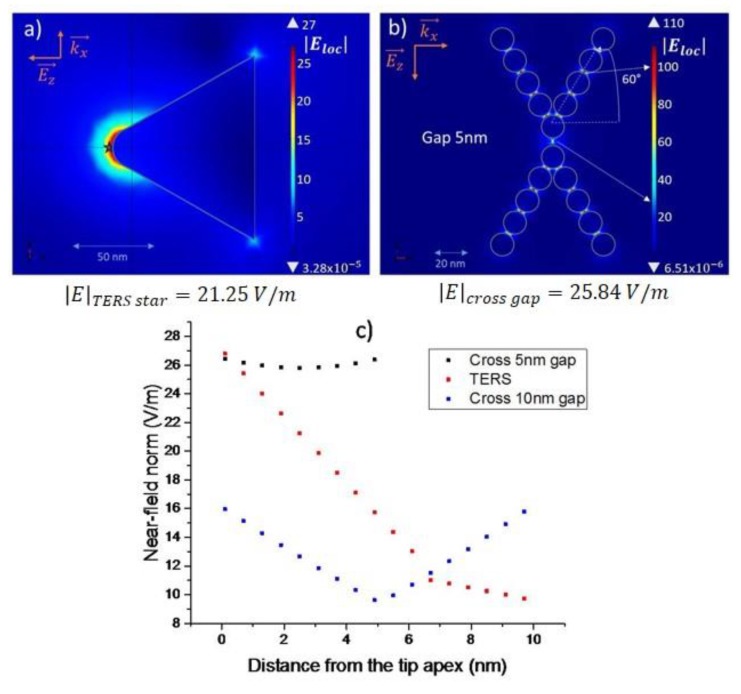
Computed near-field mappings (**a**) of a TERS tip; (**b**) a tip consisting of 9 NPs of 20 nm in diameter. The angles with respect to the normal are identical for the two structures, i.e., 60°. The respective values of the near-field norm were taken at the star point for the TERS tip, and at the white mark for the gold cross gap. (**c**) Amplitudes of the local electric field vs either the distance at the apex of the tip (TERS in red points) or the positions in the gap when the gap is only 5 nm (black) or 10 nm (blue points).

A second interesting point with this plasmonic molecule with a spatial gap with plasmonic structures from either side of the CNT 1, comes from the quasi absence of gradient of local electric field in this gap. Figure 8 shows that the plasmonic molecule presents several hot points (where the electric field multiplied by 50) localized inside the structure and not in the gap between the molecule and the atom. In this spatial spacing, the local electric field at the resonance is only multiplied by 10 but at all positions in this gap. This kind of behavior is encountered when two plasmonic molecules are separated by a small distance. The Figure 9 gives another example of one possible assembly and compares it with a gold tip usable in a TERS experiment. The exponential decrease of the amplitude of the local electric field observed for TERS configuration is then replaced by a quasi-constant electric field amplitude for a gap of 5 nm. The value of the electric amplitude obtained in this gap is equal to this one obtained at 2nm of the apex of the tip in TERS mode. Now, when the distance is increased to 10 nm the amplitude decreases and mostly a gradient of the amplitudes reappears. Of course, when there is not important gradient effect, the position of the CNT trapped in the gap does not have consequences on the measurements. This point could be important in the conception of nano-Raman sensors based on plasmonic molecules.

In our experiments, we have nano-manipulated 20nm-diameter plasmonic atoms, consequently the range of wavelength accessible by tuning of the shape and inter-distances of our plasmonic nano-structures is limited between 510 and 570 nm. To increase this range (to 700 nm), we suggest manipulating 40 or 50 nm diameter gold particles, always synthesized by a Turkevich’s way by changing synthesis conditions [21,22]. We are developing different nano-manipulations coupled with pre-micro manipulations to generate different natures of plasmonic molecules connected more or less with each other’s, in order to use different reserves of Au Nps around the area of interest. Thus, we hope to expand the possibilities of designing plasmonic molecules and without chemical direct bound between molecule and Au surfaces to be generate Raman enhancement. Finally, the possibility to design shapes and symmetry of plasmonic molecules opens the possibilities to control the physical coupling between them and other nano-objects, such as here CNTs to generate new nano-emitters based on the Raman effect.

## 5. Conclusions

This paper demonstrates the feasibility to build and to control nanostructures of Au Nps. These structures can be regarded as plasmonic molecules whose optical resonances are tuned by modifying the shape, the symmetry and the inter-particle (inter plasmonic atom) distances with the help of an AFM device coupled with an optical spectrometer. Originally, we have worked with a Raman spectrometer equipped with many excitation wavelengths in order to monitor enhanced Raman fingerprint signals of single nano-objects. For each plasmonic molecules, our approach allowed us to prospect actively their responses by changing interparticle distances and then to test several configurations with the same Au particles as well as to interpret without ambiguity the recorded changes. Indeed, when you compare plasmonic molecules constituted by different plasmonic atoms, a part of the responses can come from changes of the atoms themselves (sphericity, more or less oblong, not constant diameter, angular shape, ...) whereas this work avoids this difficulty. Let us note that our approach is applicable for nanorods too (gold or silver). For gold, this work shows that, instead of using diameters around 20 nm, an average diameter around 40 nm of plasmonic atoms will allow to tune the resonances by deforming shape of plasmonic molecule on a larger spectral range. Furthermore, for Raman applications (nano-sensor) the existence of large spacing between two plasmonic molecules allows to have a such enhanced signal as in a Tip Enhanced experiment but with a weak gradient. In this latter case, the distance between the object to be probed and the plasmonic molecules is less crucial and easier to manage than SERS and TERS experiments.

## References

[1] Willets K.A., Van Duyne R.P. (2007). Localized Surface Plasmon Resonance Spectroscoy and Sensing. Annu. Rev. Phys. Chem..

[2] Jain P.K., Huang X., El-Sayed I.H., El-Sayed M.A. (2007). Review of Some Interesting Surface Plasmon Resonance-Enhanced Properties of Noble Metal Nanoparticles and Their Applications to Biosystems. Plasmonics.

[3] Schuller J.A., Barnard E.S., Cai W., Jun Y.C., White J.S., Brongersma M.L. (2010). Plasmonics for Extreme Light Concentration andManipulation. Nat. Mater..

[4] Halas N.J., Lal S., Chang W.-S., Link S., Nordlander P. (2011). Plasmons in Strongly Coupled Metallic Nanostructures. Chem. Rev..

[5] Haran G., Chuntonov L. (2018). Artificial Plasmonic Molecules and Their Interaction with Real Molecules. Chem. Rev..

[6] Bouvrée A., D’Orlando A., Makiabadi T., Martin S., Louarn G., Mevellec J.Y., Humbert B. (2013). Nanostructured and nanopatterned gold surfaces: Application to the surface-enhanced Raman spectroscopy. Gold Bull..

[7] Punj D., Regmi R., Devilez A., Plauchu R., Moparthi S., Stout B., Bonod N., Rigneault H., Wenger J. (2015). Self-Assembled Nanoparticle Dimer Antennas for Plasmonic-Enhanced Single-Molecule Fluorescence Detection at Micromolar Concentrations. ACS Photonics.

[8] Flauraud V., Mastrangeli M., Bernasconi G.D., Butet J., Alexander D.T.L., Shahrabi E., Martin O.J.F., Brugger J. (2017). Nanoscale topographical control of capillary assembly of nanoparticles. Nat. Nanotechnol..

[9] Koenderink A.F. (2017). Single Photon Antennas. ACS Photonics.

[10] Kumar S., Johnson W., Wood C.K., Qu T., Wittenberg N.J., Otto L.M., Shaver J., Long N.J., Victora R.H., Edel J.B., Oh S.H. (2016). Template-Stripped Multifunctional Wedge and Pyramid Arrays for Magnetic Nanofocusing and Optical Sensing. ACS Appl. Mater. Interfaces.

[11] Sow I., Grand J., Lévi G., Aubard J., Félidj N., Tinguely J.C., Hohenau A., Krenn J.R. (2013). Revisiting Surface-Enhanced Raman Scattering on Realistic Lithographic Gold Nanostripes. J. Phys. Chem. C.

[12] Chuntonov L., Haran G. (2011). Trimeric Plasmonic Molecules: The Role of Symmetry. Nano Lett..

[13] Chuntonov L., Haran G. (2011). Effect of Symmetry Breaking on the Mode Structure of Trimeric Plasmonic Molecules. J. Phys. Chem. C..

[14] Prodan E., Radloff C., Halas N.J., Nordlander P. (2003). A Hybridization Model for the Plasmon Response of Complex Nanostructures. Science.

[15] Nordlander P., Oubre C., Prodan E., Li K., Stockman M.I. (2004). Plasmon Hybridization in Nanoparticle Dimers. Nano Lett..

[16] Prodan E., Nordlander P. (2004). Plasmon Hybridization in Spherical Nanoparticles. J. Chem. Phys..

[17] D’Orlando A. (2015). Nano-structuration de nanoparticules métalliques pour exaltation de champs électromagnétiques locaux en spectroscopie Raman. Ph.D. Thesis.

[18] Tong L., Li Z., Zhu T., Xu H., Liu Z. (2008). Single Gold-Nanoparticle-Enhanced Raman Scattering of Individual Single-Walled Carbon Nanotubes via Atomic Force Microscope Manipulation. J. Phys. Chem. C.

[19] Heeg S., Oikonomou A., Fernandez-Garcia R., Lehmann C., Maier S.A., Vijayaraghavan A., Reich S. (2014). Plasmon-Enhanced Raman Scattering by Carbon Nanotubes Optically Coupled with Near-Field Cavities. Nano Lett..

[20] Mueller N.S., Heeg S., Kusch P., Gaufres E., Tang N.Y.-W., Hubner U., Martel R., Vijayaraghavan A., Reich S. (2017). Plasmonic enhancement of SERS measured on molecules in carbon nanotubes. Faraday Discuss..

[21] Shi L., Buhler E., Boué F., Carn F. (2017). How does the size of gold nanoparticles depend on citrate to gold ratio in Turkevich synthesis? Final answer to a debated question. JCIS.

[22] Turkevich J., Stevenson P.C., Hillier J. (1951). A study of the nucleation and growth processes in the synthesis of colloidal gold. Discuss. Faraday Soc..

[23] Grausem J., Humbert B., Spajer M., Courjon D., Burneau A., Oswalt J. (1999). Near field Raman. J. Raman. Spectrosc..

[24] Yushanov S., Crompton J.S., Koppenhoefer K.C. MieSolution-Paper-Crompton_paper.pdf. https://cn.comsol.com/paper/download/181101/crompton_paper.pdf.

[25] Etchegoin P.G., Le Ru E.C., Meyer M. (2006). An analytic model for the optical properties of gold. J. Chem. Phys..

[26] Zhu W., Crozier K.B. (2014). Quantum Mechanical Limit to Plasmonic Enhancement as Observed by Surface-Enhanced Raman Scattering. Nat. Commun..

[27] Kataura H., Kumazawa Y., Maniwa Y., Umezub I., Susuki S., Ohtsuka Y., Achiba Y. (1999). Optical Properties of Single-Wall Carbon Nanotubes. Synth. Met..

[28] Liu K., Deslippe J., Xiao F., Capaz R.B., Hong X., Aloni S., Zettl A., Wang W., Bai X., Louie S.G., Wang E., Wang F. (2012). An atlas of carbon nanotube optical transitions. Nat. Nanotechnol..

[29] Zhang D., Yang J., Yang F., Li R., Li M., Ji D., Li Y. (2015). (n,m) Assignments and quantification for single walled carbon nanotubes on SiO_2_/Si substrates by resonant Raman spectroscopy. Nanoscale.

[30] Moura L.G., Moutinho M.V.O., Venezuela P., Fantini C., Righi A., Strano M.S., Pimenta M.C. (2014). Raman excitation profile of the G band in single-chirality carbon nanotubes. Phys. Rev. B.

